# Medical students’ perception of general practice: a cross-sectional survey

**DOI:** 10.1186/s12909-023-04064-z

**Published:** 2023-02-09

**Authors:** D. H. J. Pols, A. Kamps, J. Runhaar, G. Elshout, K. F. van Halewijn, Patrick J. E. Bindels, K. M. Stegers–Jager

**Affiliations:** 1 Department of General Practice, Erasmus MC University Medical Center Rotterdam, Rotterdam, The Netherlands; 2 Institute of Medical Education Research Rotterdam, Erasmus MC University Medical Center Rotterdam, Rotterdam, The Netherlands

**Keywords:** General practice, Medical students, Career choice, Medical education, Undergraduate

## Abstract

**Background:**

An increase in the demand for general practitioners is expected in many countries, but only a minority of medical students consider a career in general practice. More detailed and up-to-date knowledge about medical student’s perception of general practice would be helpful for efforts to encourage medical students to consider a career in general practice.

**Methods:**

We performed a cross-sectional single center survey among Dutch medical students to evaluate their perception of general practice at three different stages in their study: Ba1: first-year bachelor students; Ba3: third-year bachelor students; Ma3: third-year master students. The impact of different factors on their attitudes and perceptions was quantified. A multivariate logistic regression was performed with ‘interest in general practice’ as the outcome variable.

**Results:**

The median age for Ba1 was 18 (IQR: 18–19) and 71.5% were female, for Ba3 the median age was 20 (IQR: 20–21) and 70.6% were female and for Ma3 the median age was 25 years (IQR: 24–26) and 73.3% were female. On average, 31.2% of the respondents had a migration background. The mean response rate for this study was 77.1%. Of the participating Ba1 students (*n* = 340) only 22.4% considered working as a GP after medical school; for Ba3 students (*n* = 231) this percentage was 33.8%, and for Ma3 students (*n* = 210) it was significantly higher at 70.5%; in the final multivariate model this corresponded to an odds ratio (OR) of 4.3 (95%-CI:2.6–6.9) compared to Ba1 students. The strongest predictor in the final model was the opinion that general practice provides a pleasant working environment (OR 9.5; 95%-CI: 6.2–14.5).

**Conclusion:**

This study showed that multiple factors are significantly related to medical students’ interest in general practice. Although students believed that general practice does not have a high status within the medical profession, they acknowledged the social importance and the pleasant working environment of general practice. Knowledge obtained in this study can be used when designing a medical curriculum or a general practice course.

**Supplementary Information:**

The online version contains supplementary material available at 10.1186/s12909-023-04064-z.

## Background

Access to high-quality primary care is essential for a well-functioning healthcare system. Countries with strong primary care systems report lower health care costs, reduced health inequalities and higher quality of care [1]. An increase in the demand for general practitioners is expected in many countries [2], but only a minority of medical students consider a career in general practice [3–6]. To encourage medical students to pursue general practice as their postgraduate specialization, a good understanding of medical students’ perception of general practice is helpful. The international literature has already identified multiple factors that influence these perceptions, such as personal characteristics of the student (e.g. age, sex, socioeconomic status, personal experience with general practice), educational environment (e.g. learning experience, opinion of fellow students or teachers) and the student’s perception of general practice (e.g. workload, career possibilities, income) [7–12]. But can we quantify the impact of such factors so that smarter choices can be made, e.g. when designing a general practice course, to encourage more medical students to pursue general practice as their postgraduate specialization?

All university medical centres in the Netherlands have a department of general practice, that teaches bachelor students clinical reasoning and provides training in basic clinical skills. In this way, medical students are already exposed to general practice during the first years of their study, although only to a moderate extent. During their master’s programme, all students do a mandatory internship in a general practice clinic; in some universities, this is preceded by a general practice course.

From a questionnaire study among Dutch medical students in 2019, we learned that 7% of the bachelor students were considering a career in general practice, a percentage that increased to 20% among master students [3]. These results are in agreement with the international literature, which shows that medical students’ interest in general practice increases as they progress through their study and that this increase was correlated with the level of exposure they had to general practice during their undergraduate training [13, 14].

More detailed and up-to-date knowledge about medical student’s perception of general practice would be helpful for efforts to encourage medical students to consider a career in general practice. We performed a cross-sectional survey among medical students to evaluate their perception of general practice at three different stages in their study.

## Methods

### Participants

This study was conducted at Erasmus MC University Medical Center Rotterdam in the Netherlands. Compared with other Dutch medical schools this school has a relatively large number of ethnic minority students. The curriculum of this school consists of two parts.

First part: a bachelor’s programme of three years, which mainly focuses on theory. The integrated and theme-oriented bachelor curriculum is divided into three thematic blocks per year. Each thematic block consists of 2–3 sub-blocks. The bachelor program includes two types of examinations: written examinations and clinical skills examinations. The written theoretical knowledge examinations are further divided into block tests at the end of each thematic sub-block and a clinical problem solving test at the end of each year of the Bachelor degree course. In these years GPs provide students with clinical reasoning and clinical skills courses. GPs are also responsible for the clinical problem solving test in Ba1 and Ba2, as well as for the majority of the clinical skills examinations.

Second part: a master’s programme of three years, focusing on gaining clinical experience. It consists of thematic education (‘just in time education’ before each discipline-specific internship), a master research (either before or after the discipline-specific internships), and 12 discipline-specific internships. Internships take place in a fixed sequence and include the following: internal medicine (10 weeks), surgery (10 weeks), pediatrics (5 weeks), psychiatry (5 weeks), neurology (5 weeks), gynecology (5 weeks), dermatology (3 weeks), ear, nose and throat surgery (3 weeks), ophthalmology (3 weeks), general practice (6 weeks) and social medicine (3 weeks). After these compulsory internships, students have the opportunity to choose an internship of interest for the remaining 18 weeks of their study (semi-doctor internship).

The participants for this study were medical students in the first year (Ba1) or third year (Ba3) of their bachelor training (academic year 2020–2021), and third-year master students (Ma3) who had finished their internship in general practice between November 2020 and June 2021. The first-year students (Ba1) can be considered as a reference group, with hardly any exposure to general practice so far. In the third year (Ba3), the students have a better understanding of medicine, but do not have clinical experience yet. By the sixth year of medical school (Ma3), the students have gained a lot of clinical experience, and have also finished a two-week course in general practice and a consecutive six-week internship in a general practice clinic.

### Student questionnaire

The methods for our study were based on a protocol published by Alonso-Coello et al. [15]. The measurement instrument was a specifically designed and validated three-part questionnaire covering 1) perceptions about general practice, 2) perceptions of preparation for general practice in the medical programme and 3) expectations and preferences for future career choices [15]. Demographic information was collected at the end of the questionnaire. For this study we focused primarily on the first part of the questionnaire. Eight statements were analysed considering students’ perceptions about the social and scientific status of general practice, as well as six items studying the importance of factors that could have influenced these perceptions and finally six items on feedback within the medical school about general practice. All multiple-choice items used a six-point Likert scale (1 = totally disagree/none – > 6 = totally agree/much), with some additional open-ended questions. The original English questionnaire was translated into Dutch according to academic standards (forward and backward translation by native speakers). The original demographic information section included year of study, gender, size of the community where pre-university studies were completed, family or friends working in health care, participation in voluntary service activities, and participation in university exchange programmes. Additional student characteristics that might be related to medical students’ perception of medical specializations, were added based on an expert meeting. We included the following additional questions in the questionnaire: What is your age? What is your country of birth? And what is the country of birth of your parents? In what language do you communicate with your parents? Do you think you have a good idea of the profession of a general practitioner? The questionnaire used in this study contained 105 items (Appendix 1). 45 items were used for the analysis for this study.

### Data collection

Data were collected in the second quarter of the academic year 2020–2021 for Ba1 and Ba3. For Ma3 data were collected on the last day of their internship in a general practice clinic. The questionnaire was filled in online because of the COVID pandemic, as part of online training sessions in clinical reasoning (Ba1 and Ba3) and online reflection sessions in the context of their internship (Ma3). Participation was voluntary. The students did not receive any incentive for completing the questionnaire. EvaSys Survey Automation Suite v7.1 was used to collect the data.

### Data analysis

All questions with a six-point Likert scale were recoded into dichotomized variables: scores of 1–4 were considered negative, and 5 or 6 positive (Additional file 2). Migration background was defined as one or both parents born outside of the Netherlands. Descriptive analyses were performed, with categorical data presented in percentages and numerical data summarized by the median plus interquartile range to take into account skewed distributions. Baseline characteristics were tested for differences between the three study years (Ba1, Ba3, Ma3) using the nonparametric Pearson’s chi square test (χ^2^) and Kruskal Wallis test, whereby a *P*-value < 0.05 was considered to be statistically significant.

To assess differences in medical students’ perceptions of general practice in relation to the level of exposure, the dichotomized variables regarding perceptions of general practice (questions 2.1–2.20) were compared between study years, using χ^2^ tests. Next, Bonferroni-adjusted pairwise Z-tests were performed as post-hoc analyses for the variables that showed overall statistically significant differences, to determined which study years differed from one another.

Finally, we performed a multivariate logistic regression analysis with the dichotomized ‘interest in general practice’ as the outcome variable and multiple predictor variables. The predictor variables initially used were student characteristics (‘age’, ‘sex’, ‘study year’, ‘migration background’ and ‘parent(s) is/are a physician’) and variables that were related to their perceptions of general practice (questions 2.1–2.20, appendix 1). After running the full model, the least significant variables were eliminated in a backward step-wise selection with a *P*-value threshold of < 0.1. This resulted in an easy-to-interpret and more generalizable model, containing only the predictor variables that contributed most to ‘interest in general practice’ across all study years.

All statistical analyses were performed using IBM SPSS statistics version 25.

### Ethical approval

Institutional review board approval of the study was granted by the Medical Ethics Committee Erasmus MC of Rotterdam, the Netherlands (2020–0630) in accordance with the Dutch Medical Research with Human Subjects law. Informed consent was obtained from the study participants. Participation in this anonymous questionnaire was voluntary and participants could refuse to participate or discontinue participation at any time without penalty. The anonymous questionnaires could not be traced back to individuals. Data was stored on secure servers and only people involved in this study had access to it. All methods were performed in accordance with relevant guidelines and regulations.

## Results

### Student characteristics

The total number of questionnaires returned was 781 (Table 1). The response rates for Ba1, Ba3 en Ma3 were 82%, 58% and 90% respectively. There were no missing items. The median age for Ba1 was 18 (IQR: 18–19) and 71.5% were female, for Ba3 the median age was 20 (IQR: 20–21) and 70.6% were female and for Ma3 the median age was 25 years (IQR: 24–26) and 73.3% were female. On average, 31.2% of the respondents had a migration background. 12.2% of the medical student had parent(s) who worked as a physician. Other than age, there were no significant differences between the three groups. The characteristics of the students in this study are in accordance with the characteristics of all the registered students at our university [16], making selection bias unlikely.

**Table 1:** Baseline characteristics

	Ba 1 (*n* = 340)	Ba 3 (*n* = 231)	Ma 3 (*n* = 210)	*P*-value
Age, median (IQR)	18 (18–19)	20 (20–21)	25 (24–26)	0.000
Gender, % female	71.5	70.6	73.3	0.806
Migration background (% with migration background)	34.4	29.0	28.6	0.243
Population size of the area where you went to school				0.595
< 10,000 inhabitants (%)	19.4	22.1	17.6	
10,000–300,000 inhabitants (%)	59.7	55.0	57.1	
> 300,000 inhabitants (%)	20.9	22.9	25.2	
Parent(s) is/are physician(s)				0.861
No (%)	88.8	88.3	85.7	
Yes, excl. G.P (%)	6.8	7.4	8.6	
Yes, G.P. (%)	4.4	4.3	5.7	
Relatives/friend working in primary care (%)	42.1	39.0	38.1	0.601

### Perceptions about general practice

**Fig. 1:**
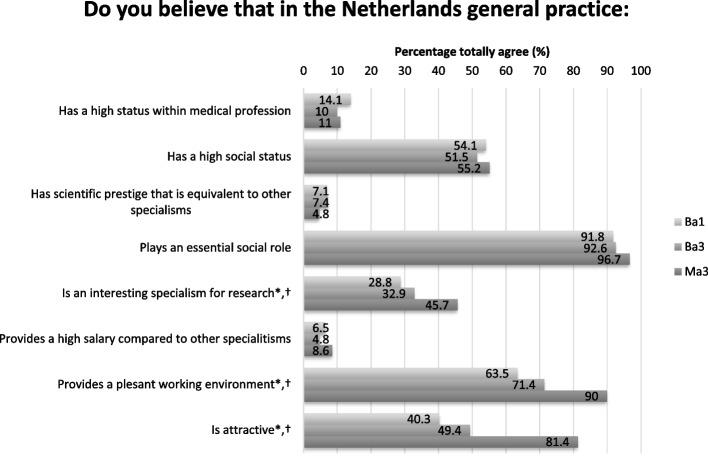
Perceptions about general practice. *Statistically significant *P* < 0.05: Ba1 vs Ma3; † Statistically significant *P* < 0.05: Ba3 vs Ma3. Ba1: first-year bachelor students; Ba3: third year bachelor students; Ma3: third-year master students who had finished their internship in general practice

Overall, only a minority of the medical students believed that general practice had 1) a high status within the medical profession (12.1%), 2) a scientific prestige equivalent to that of other specialism (6.6%), and 3) a high salary compared to other specialisms (6.6%). Just over half (53.6%) of the students believed that general practice had a high social status. The vast majority (93.4%) agreed that general practice has an essential role in Dutch society. Some perceptions differed significantly between study years. Ma3 students scored higher on specific perceptions compared to bachelor students. These higher scores were seen for students believing general practice to be 1) an interesting specialism for research (Ba1:28.2% versus Ma3:45.7%), 2) an attractive working domain (Ba1: 40.3% versus Ma3:81.4%), and 3) a pleasant working environment (Ba1:63.5% versus Ma3:90%). Perceptions about general practice are presented for all three groups separately in Fig. 1.

### Self-reported factors associated with interest in general practice

**Fig. 2:**
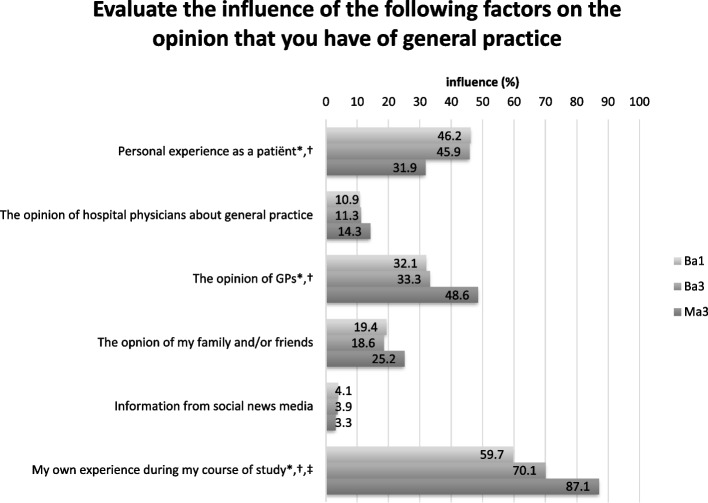
Self-reported factors influencing interest in general practice. *Statistically significant *P* < 0.05: Ba1 vs Ma3; † Statistically significant *P* < 0.05: Ba3 vs Ma3; ‡ Statistically significant *P* < 0.05: Ba1 vs Ba3. Ba1: first-year bachelor students; Ba3: third year bachelor students; Ma3: third-year master students who had finished their internship in general practice

A majority of the students reported that their own experience with general practice during the medical school course substantially impacted their interest in general practice (Fig. 2). This was true for 59.7% of the Ba1 students and this percentage was significantly higher for Ma3 (87.1%). The students’ personal experience as a patient also played an important role according to nearly half of Ba1 students (46.2%) and Ba3 students (45.9%), but this proportion was significantly lower in the Ma3 group (31.9%). The opinions of GPs about general practice as given during courses, had a significantly greater impact on the Ma3 group (48.6%) compared to the Ba groups (Ba1: 32.1%; Ba3: 33.3%). Information from social media had limited influence across the study years (3.8%).

### Influencing factors from regression analysis

Whereas only 22.4% of the Ba1 students considered working as a GP after medical school, for Ba3 students this percentage was 33.8%, and for Ma3 students it increased significantly to 70.5%. The impact of factors that could influence medical students to consider a career in general practice was studied in a regression analysis. In the final model, 14 variables correlated with an interest in general practice, and improved the pre-test probability for correct classification from 61 to 81% (Table 2).

**Table 2:** Multivariate regression with the binary dependent variable ‘I would consider postgraduate GP training’. Odds ratios (ORs) with corresponding 95% confidence intervals (CIs) are listed for the answer-categories for the variables that were maintained in the analysis after backward selection. For variables with three answer-categories, the reference answer-category is given as well

Variable, answer category	OR	95% CI Lower	95% CI Upper
Study year, Ba1 (reference)			
Study year, Ba3*	1.7	1.1	2.6
Study year, Ma*	4.3	2.6	6.9
Migration background, yes	0.7	0.5	1.0
Sex, female*	1.7	1.1	2.7
Interesting specialty for research, yes*	1.7	1.2	2.6
Provides pleasant working environment, yes*	9.5	6.2	14.5
Influence of GP’s opinion, very much*	1.9	1.3	2.9
Influence of opinion of family/friends, very much*	1.7	1.1	2.7
Influence of own experience during study, very much	1.5	0.9	2.3
Comments of teachers, none (reference)			
Comments of teachers, negative comments*	1.7	1.1	2.7
Comments of students, none (reference)			
Comments of students, negative comments	0.7	0.4	1.0

^*^Statistically significant on a *P* < 0.05 level

Students who were further in their training were significantly more likely to consider working in general practice than Ba1 students (Ma group: OR: 4.3, 95%-CI:2.6–6.9). Having a migration background seemed to reduce this interest by a factor of 0.7 (95%-CI: 0.5–1.0), although this was not significant. Remarkably, negative comments by teachers about general practice were associated with greater interest among students in general practice (OR:1.7, 95%-CI:1.1–2.7). The largest effect on interest was found for the opinion that general practice provides a pleasant working environment. If this was the belief of a medical student, the odds for considering general practice as a career were 9.5 (95%-CI: 6.2–14.5) times greater than if the student did not hold this opinion.

## Discussion

### Summary

We performed a cross-sectional survey study among medical students to evaluate their perception of general practice at three different stages in their education, and quantified the impact of different factors on their perceptions. Although students believed that general practice does not have a high status within the medical profession, they acknowledged the social importance of general practice. Interest in a career in general practice differs between Ba1 students (22% have an interest) and Ma3 students (71%). If a student believed that general practice provides a pleasant working environment, the chance of considering general practice as their postgraduate specialization was 9.5 times greater than id they did not believe this. Only 7% of the students believed that general practice has a scientific prestige that is equivalent to other specialisms.

### Strengths and limitations

To our knowledge, this study is the first of its kind to quantify the impact of different factors on the interest in general practice among students who have been exposed to different levels of general practice and related courses. The strengths of our study include the large number of participating students at different stages in their medical school training and the use of an extensive and validated questionnaire. Study limitations include the cross-sectional design of this study. No causal statements can be made. Since a randomized controlled trial is not feasible, a prospective longitudinal cohort study may provide further insight in the factors influencing the students’ perceptions about general practice. Choosing a single centre design was a pragmatic choice. Since most medical curricula in the Netherlands are broadly structured in the same way, we expect that the results of this study can be interpreted more broadly. We are currently looking at designing a multi-centre study. Ba3 students had a lower response rate. We don’t have an explanation for this observation, but based on the baseline characteristics of this group, there were now signs for selection bias. Finally, in this study the interest in general practice was measured, but not the actual enrolment in the post graduate training for general practice.

Data was collected during the COVID peak, however we did not obtain any signs that this will have substantially influenced the results of this study.

### Comparison with existing literature

Having enough GPs is of paramount importance to keep healthcare systems healthy and working. This study showed a greater interest in general practice in master students compared to first and third year bachelor students. This is presumably due in part to the two-week general practice course and a consecutive six-week internship in the final year of medical school. International research demonstrates that medical students’ attitudes towards general practice improve after completing a course in general practice [11, 15, 17, 18] or an internship [19]. We have been questioning whether our GP course comes too late in our curriculum. However, Lopez-Garcia showed that at the end of undergraduate training, there are no significant differences between students who have taken a GP-course in the second year and those who took such a course in the sixth year regarding their postgraduate interest in general practice [20]. The fact that 71% of Ma3 students in this survey were interested in general practice probably helps explain the 30% enrolment by our graduate doctors in the postgraduate general practice training, while the average for the Netherlands is 22% [21]. With the knowledge obtained in this study, we will try to further improve these numbers.

Only 7% of the students believed that general practice has a scientific prestige that is equivalent to other specialisms, although over the course of the study a significantly increasing number of students believe it is an interesting specialism for research. The literature suggests that students believe that working in general practice limits their research opportunities [22]. Providing medical students with more primary care studies during e.g. evidence based medicine-courses could help to improve the scientific prestige of general practice among students. According to our study, this increased the interest in general practice by a factor of 1.7. Currently, our students are hardly exposed to primary care research during the bachelor programme. Another way to improve the scientific prestige of general practice research is to encourage more medical students to choose primary care studies for their master thesis.

Having a migration background seemed to reduce this interest by a factor of 0.7 (95%-CI: 0.5–1.0), although this was not a significant result. A review published by Levaillant et al. [23] concluded that “attractiveness of some specialties varied depending on origin country”. General practice attended the third place (10,3%) in occidental countries (North America, European Union, Australia and New-Zealand) and sixth (5,3%) in non-occidental countries (other countries). This observation is in line with the negative odds ratio found in this study.

## Conclusions

This study showed that the interest in general practice is higher in master students than in bachelor students. It seems relevant to introduce courses on general practice during medical training so that students can experience that general practice is an attractive specialism and provides a pleasant working environment. This may help generate an interest in general practice. Also, exposing students to more primary care research in medical school could also help to increase the interest in general practice even further and to improve the scientific prestige of general practice among students. Future research may provide further insight in the factors influencing the students’ perceptions about general practice. A multi-centre prospective longitudinal cohort design would be the next step.

## Supplementary Information

**Additional file 1.**

**Additional file 2: Appendix 2.** Variable names and definitions.

## Data Availability

The datasets used and/or analysed during the current study are available from the corresponding author on reasonable request.
