# Supplementary material for: Medical students’ perception of general practice: a cross-sectional survey
Source: BMC Med Educ. 2023 Feb 9;23:103. doi: 10.1186/s12909-023-04064-z (PMC9912627; doi:10.1186/s12909-023-04064-z)
Supplement: Supplementary file 1 — Additional file 1. [file 12909_2023_4064_MOESM1_ESM.pdf]

EvaSys	Onderzoek huisartsgeneeskunde [Copy]	Electric Paper

Mark as shown: ☐ ☒ ☐ ☐ ☐ Please use a ball-point pen or a thin felt tip. This form will be processed automatically.

Correction: ☐ ■ ☐ ☒ ☐ Please follow the examples shown on the left hand side to help optimize the reading results.

1. Studiejaar

1.1 In welk jaar van de studie geneeskunde zit je op dit moment?
☐ Ba1 ☐ Ba2 ☐ Ba3
☐ Ma1 ☐ Ma2 ☐ Ma3

2. Opvattingen over huisartsgeneeskunde

Geef je mening over onderstaande stellingen. Geloof jij dat huisartsgeneeskunde in Nederland:

2.1	Een hoge status heeft binnen de medische wereld	Totaal mee oneens	☐	☐	☐	☐	☐	☐	Totaal mee eens
2.2	Een hoge maatschappelijke status heeft	Totaal mee oneens	☐	☐	☐	☐	☐	☐	Totaal mee eens
2.3	Een wetenschappelijk aanzien heeft dat gelijk is aan andere specialismen	Totaal mee oneens	☐	☐	☐	☐	☐	☐	Totaal mee eens
2.4	Een essentiële maatschappelijke rol speelt	Totaal mee oneens	☐	☐	☐	☐	☐	☐	Totaal mee eens
2.5	Een interessant specialisme is voor wetenschappelijk onderzoek	Totaal mee oneens	☐	☐	☐	☐	☐	☐	Totaal mee eens
2.6	Een hoog salaris biedt ten opzichte van andere specialismen	Totaal mee oneens	☐	☐	☐	☐	☐	☐	Totaal mee eens
2.7	Een aangename werkomgeving biedt	Totaal mee oneens	☐	☐	☐	☐	☐	☐	Totaal mee eens
2.8	Een aantrekkelijk werkdomein is	Totaal mee oneens	☐	☐	☐	☐	☐	☐	Totaal mee eens

Hoeveel invloed hebben de volgende factoren op jouw mening over huisartsgeneeskunde?

2.9	Mijn persoonlijke ervaring als patiënt	Geen	☐	☐	☐	☐	☐	☐	Veel
2.10	De mening van ziekenhuisspecialisten over huisartsgeneeskunde	Geen	☐	☐	☐	☐	☐	☐	Veel
2.11	De mening van huisartsen	Geen	☐	☐	☐	☐	☐	☐	Veel
2.12	De mening van mijn familie/vrienden	Geen	☐	☐	☐	☐	☐	☐	Veel
2.13	Informatie van sociale media	Geen	☐	☐	☐	☐	☐	☐	Veel
2.14	Mijn eigen ervaring gedurende de studie geneeskunde	Geen	☐	☐	☐	☐	☐	☐	Veel

2.15 Heb je gedurende de studie geneeskunde opmerkingen gehoord over huisartsgeneeskunde? ☐ Nee ☐ Ja

Evalueer de opmerkingen die je hebt gehoord van:

2.16	Ziekenhuisspecialisten	Heel negatief	☐	☐	☐	☐	☐	☐	Heel positief
2.17	Huisartsen	Heel negatief	☐	☐	☐	☐	☐	☐	Heel positief
2.18	Docenten	Heel negatief	☐	☐	☐	☐	☐	☐	Heel positief
2.19	Arts-assistenten	Heel negatief	☐	☐	☐	☐	☐	☐	Heel positief

DRAFT

2. Opvattingen over huisartsgeneeskunde [Continue]

2.20	Medestudenten	Heel negatief	☐	☐	☐	☐	☐	☐ Heel positief

3. Huisartsgeneeskundig onderwijs tijdens de studie geneeskunde

Geef je mening over de volgende stellingen:

3.1	Er zijn voldoende redenen om theoretisch en praktisch onderwijs over huisartsgeneeskunde te verplichten voor de studie geneeskunde	Totaal mee oneens	☐	☐	☐	☐	☐	☐ Totaal mee eens

Onderwijs in huisartsgeneeskunde zou verplicht moeten zijn omdat:

3.2	Huisartsgeneeskunde een specifieke wetenschappelijke setting is	Totaal mee oneens	☐	☐	☐	☐	☐	☐ Totaal mee eens
3.3	Huisartsgeneeskunde een veelvoorkomende beroepskeuze is	Totaal mee oneens	☐	☐	☐	☐	☐	☐ Totaal mee eens
3.4	Huisartsgeneeskunde bijdraagt aan de verbetering van de zorg	Totaal mee oneens	☐	☐	☐	☐	☐	☐ Totaal mee eens
3.5	Huisartsgeneeskunde een centraal onderdeel is van de gezondheidszorg	Totaal mee oneens	☐	☐	☐	☐	☐	☐ Totaal mee eens

---

Onderwijs in huisartsgeneeskunde zou:

3.6	Geïntegreerd moeten worden met bestaande onderwijsblokken	Totaal mee oneens	☐	☐	☐	☐	☐	☐ Totaal mee eens
3.7	Een specifiek onderwijsblok moeten zijn	Totaal mee oneens	☐	☐	☐	☐	☐	☐ Totaal mee eens
3.8	Moeten voldoen aan beide bovenstaande opties	Totaal mee oneens	☐	☐	☐	☐	☐	☐ Totaal mee eens

---

3.9	Het onderwijs in huisartsgeneeskunde moet altijd worden onderwezen door huisartsen	Totaal mee oneens	☐	☐	☐	☐	☐	☐ Totaal mee eens

3.10 In welk jaar van de studie geneeskunde zou dit onderwijs moeten beginnen?

☐ Ba1 ☐ Ba2 ☐ Ba3
☐ Ma1 ☐ Ma2 ☐ Ma3

Beoordeel het belang van de volgende bijdrages van huisartsgeneeskunde aan de studie geneeskunde:

3.11	Communicatie/arts-patiënt relatie	Geen	☐	☐	☐	☐	☐	☐ Veel
3.12	Preventie en gezondheidsbevordering	Geen	☐	☐	☐	☐	☐	☐ Veel
3.13	Toepassing van het biopsychosociale model in de zorg	Geen	☐	☐	☐	☐	☐	☐ Veel
3.14	Familiegerichtheid van zorg	Geen	☐	☐	☐	☐	☐	☐ Veel
3.15	Maatschappelijke zorg	Geen	☐	☐	☐	☐	☐	☐ Veel
3.16	Medische zorg voor de meest voorkomende problemen	Geen	☐	☐	☐	☐	☐	☐ Veel
3.17	Zorg gedurende het gehele leven	Geen	☐	☐	☐	☐	☐	☐ Veel
3.18	Spoedeisende zorg	Geen	☐	☐	☐	☐	☐	☐ Veel
3.19	Klinische epidemiologie	Geen	☐	☐	☐	☐	☐	☐ Veel
3.20	Teamwork	Geen	☐	☐	☐	☐	☐	☐ Veel
3.21	Medische ethiek	Geen	☐	☐	☐	☐	☐	☐ Veel
3.22	Wetenschappelijk onderzoek	Geen	☐	☐	☐	☐	☐	☐ Veel
3.23	Samenwerking met andere disciplines (onderwijs, sociaal, ….)	Geen	☐	☐	☐	☐	☐	☐ Veel

DRAFT

3. Huisartsgeneeskundig onderwijs tijdens de studie geneeskunde [Continue]

3.24 Geef jouw mening over het nut van het coschap van de huisartsgeneeskunde. (Indien je nog geen coschap huisartsgeneeskunde hebt gevolgd, vul dan nvt in.) Geen ☐ ☐ ☐ ☐ ☐ ☐ Veel ☐ Nvt

3.25 Gedurende welk jaar / jaren van de studie geneeskunde moet het coschap plaatsvinden?
☐ Ba1 ☐ Ba2 ☐ Ba3
☐ Ma1 ☐ Ma2 ☐ Ma3

3.26 Welk percentage van de totale studie geneeskunde moet volgens jou worden besteed aan huisartsgeneeskunde?
☐ <10% ☐ 10-25% ☐ 26-50%
☐ 51-75% ☐ 76-90% ☐ >90%

4. Verwachtingen en voorkeuren

Evalueer jouw mate van interesse in het werken in een van de volgende specialismen na de studie geneeskunde.

4.1	Beschouwende ziekenhuis specialismen (bijvoorbeeld interne geneeskunde, neurologie etc.)	Geen ☐	☐	☐	☐	☐	☐	Veel
4.2	Snijdende ziekenhuis specialismen (bijvoorbeeld chirurgie, urologie etc.)	Geen ☐	☐	☐	☐	☐	☐	Veel
4.3	Huisartsgeneeskunde	Geen ☐	☐	☐	☐	☐	☐	Veel
4.4	Kindergeneeskunde	Geen ☐	☐	☐	☐	☐	☐	Veel
4.5	Gynaecologie	Geen ☐	☐	☐	☐	☐	☐	Veel
4.6	Psychiatrie	Geen ☐	☐	☐	☐	☐	☐	Veel
4.7	Dermatologie	Geen ☐	☐	☐	☐	☐	☐	Veel
4.8	Oogheelkunde	Geen ☐	☐	☐	☐	☐	☐	Veel
4.9	Keel-, neus- en oorheelkunde	Geen ☐	☐	☐	☐	☐	☐	Veel
4.10	Sociale geneeskunde	Geen ☐	☐	☐	☐	☐	☐	Veel
4.11	Laboratorium	Geen ☐	☐	☐	☐	☐	☐	Veel
4.12	Radiologie	Geen ☐	☐	☐	☐	☐	☐	Veel
4.13	Onderzoek	Geen ☐	☐	☐	☐	☐	☐	Veel
4.14	Onderwijs	Geen ☐	☐	☐	☐	☐	☐	Veel

Beoordeel welke kenmerken van een specialisme belangrijk zullen zijn voor je specialisatiekeuze na je studie geneeskunde.

4.15	Biedt een breed aanbod van klinische problemen	Niet ☐	☐	☐	☐	☐	☐	Zeer
4.16	Maakt het mogelijk een breed scala aan patiënten van uiteenlopende leeftijden te zien	Niet ☐	☐	☐	☐	☐	☐	Zeer
4.17	Concentreert zich op een specifiek aandachtsgebied	Niet ☐	☐	☐	☐	☐	☐	Zeer
4.18	Maakt het mogelijk direct resultaat te zien van jouw professioneel handelen	Niet ☐	☐	☐	☐	☐	☐	Zeer
4.19	Heeft wetenschappelijk aanzien	Niet ☐	☐	☐	☐	☐	☐	Zeer
4.20	Vereist veel professionele toewijding	Niet ☐	☐	☐	☐	☐	☐	Zeer
4.21	De mate waarin het volgen van de specialistenopleiding inspanning vereist	Niet ☐	☐	☐	☐	☐	☐	Zeer
4.22	Biedt mogelijkheid tot een hoog salaris	Niet ☐	☐	☐	☐	☐	☐	Zeer
4.23	Maakt het mogelijk een hechte relatie met patiënten op te bouwen	Niet ☐	☐	☐	☐	☐	☐	Zeer
4.24	Maakt goede arbeidsvoorwaarden en kwaliteit van leven mogelijk	Niet ☐	☐	☐	☐	☐	☐	Zeer

4. Verwachtingen en voorkeuren [Continue]

4.25 Mocht je in de toekomst als huisarts gaan werken, hoe tevreden zou je zijn? Helemaal niet tevreden ☐ ☐ ☐ ☐ ☐ ☐ Heel tevreden

4.26 Had je bij het begin van de studie geneeskunde al een duidelijk beeld over welke specialisatie je zou kiezen? ☐ Nee ☐ Ja

4.27 Is jouw voorkeur voor een specialisatie veranderd gedurende de studie geneeskunde ? ☐ Nee ☐ Ja

Beoordeel in welke mate de volgende factoren jouw keuze voor een specialisatie hebben veranderd.

4.28 Meningen en informatie van zorgprofessionals	Niet	☐	☐	☐	☐	☐	☐	Zeer
4.29 Meningen en informatie van docenten	Niet	☐	☐	☐	☐	☐	☐	Zeer
4.30 Meningen en informatie van medestudenten	Niet	☐	☐	☐	☐	☐	☐	Zeer
4.31 Meningen en informatie van familieleden en vrienden	Niet	☐	☐	☐	☐	☐	☐	Zeer
4.32 Informatie van sociale media	Niet	☐	☐	☐	☐	☐	☐	Zeer
4.33 Theoretische inhoud van het curriculum	Niet	☐	☐	☐	☐	☐	☐	Zeer
4.34 Praktische inhoud van het curriculum	Niet	☐	☐	☐	☐	☐	☐	Zeer

4.35 Heb je onderwijs gehad met betrekking tot huisartsgeneeskunde? ☐ Nee ☐ Ja

4.36 Beoordeel je tevredenheid over dit onderwijs Helemaal niet tevreden ☐ ☐ ☐ ☐ ☐ ☐ Heel tevreden

4.37 Heb je al een coschap in de huisartsgeneeskunde gehad? ☐ Nee ☐ Ja

4.38 Beoordeel je tevredenheid over dit coschap Helemaal niet tevreden ☐ ☐ ☐ ☐ ☐ ☐ Heel tevreden

4.39 Gedurende de studie geneeskunde, zou je zeggen dat jouw interesse in huisartsgeneeskunde is: ☐ Afgenomen ☐ Niet veranderd ☐ Toegenomen

5. Persoonlijke informatie

5.1 Geslacht ☐ Man ☐ Vrouw

5.2 Wat is je leeftijd?

[ ][ ]

5.3 In welk land ben je geboren? ☐ Nederland ☐ Anders

5.4 Geboorteland

[ ]

5.5 Hoeveel inwoners heeft de plaats, waar je het grootste gedeelte van je jeugd hebt gewoond? ☐ < 10.000 inwoners ☐ 10.000 - 300.000 inwoners ☐ > 300.000 inwoners

5.6 In welk land is je vader geboren? ☐ Nederland ☐ Anders

5.7 Geboorteland vader:

[ ]

5.8 In welk land is je moeder geboren? ☐ Nederland ☐ Anders

5. Persoonlijke informatie [Continue]

5.9 Geboorteland moeder

[ ]

5.10 In welke taal communiceer je met je ouders? ☐ Nederlands ☐ Anders

5.11 Taal

[ ]

5.12 Is een van je ouders/verzorgers werkzaam (geweest) als arts? ☐ Nee ☐ Ja

5.13 Is een van je ouders/verzorgers werkzaam (geweest) als huisarts? ☐ Nee ☐ Ja

5.14 Zijn er gezinsleden/vrienden werkzaam binnen de eerstelijnszorg (huisarts, verpleegkundige, verloskundige, doktersassistent)? ☐ Nee ☐ Ja

5.15 Heeft een van je ouders/verzorgers een opleiding in het hoger onderwijs (HBO of universiteit of gelijkwaardige buitenlandse opleiding) afgerond? ☐ Nee ☐ Ja

5.16 Heb je ooit vrijwilligerswerk gedaan voor een organisatie gedurende langer dan 1 maand? ☐ Nee ☐ Ja

5.17 Heb je ooit deelgenomen aan een universitair uitwisselingsprogramma? ☐ Nee ☐ Ja

5.18 In welk jaar van je studie? ☐ 1 ☐ 2 ☐ 3 ☐ 4 ☐ 5 ☐ 6

5.19 In welk kalenderjaar ben je met geneeskunde begonnen?

[ ][ ][ ][ ]

5.20 Vind je dat je een goed beeld hebt van het beroep huisarts? ☐ Nee ☐ Ja

---

Hartelijk dank voor het invullen van de vragenlijst en deelname aan het onderzoek!
