# Supplementary material for: Medical students’ perception of general practice: a cross-sectional survey
Source: BMC Med Educ. 2023 Feb 9;23:103. doi: 10.1186/s12909-023-04064-z (PMC9912627; doi:10.1186/s12909-023-04064-z)
Supplement: Supplementary file 2 — Additional file 2: Appendix 2. Variable names and definitions. [file 12909_2023_4064_MOESM2_ESM.pdf]

Appendix 2. Variable names and definitions.

Variable name	Definition or question asked	Levels
**Age**	What is your age in years?	
**Sex**	What is your sex?	1. Female<br>2. Male
**Study year**	What year of your medicine study are you currently in?	1. Bachelor 1st year<br>2. Bachelor 3rd year<br>3. Master
**Migration background**	In which country were your mother and father born?	1. Netherlands (no migration background)<br>2. One or both of parents outside of the Netherlands (migration background)
**Parent(s) is/are physician**	Is one of your parents a physician? If yes, is he or she a general practitioner?	1. No physician<br>2. Physician (other than general practitioner)<br>3. General practitioner
**Interest in general practice**	Evaluate your level of interest in working in general practice after studying medicine	1. No or little interest<br>2. Very interested
	Do you believe that general practice in the Netherlands:	1. Totally disagree to neutral<br>2. (Totally) agree
**2.1**	Has a high status within the medical world	" "
**2.2**	Has a high social status	" "
**2.3**	Has a scientific reputation equal to other specialisms	" "
**2.4**	Plays an essential social role	" "
**2.5**	Is an interesting specialism for scientific research	" "
**2.6**	Offers a high salary compared to other specialisms	" "
**2.7**	Provides a pleasant working environment	" "
**2.8**	Is an attractive work domain	" "
	How much influence do the following factors have in your opinion about general practice?	1. No or little influence<br>2. A lot of influence
**2.9**	My personal experience as a patient	" "
**2.10**	The opinion of hospital specialists regarding general medicine	" "
**2.11**	The opinion of general practitioners	" "
**2.12**	The opinion of my family/friends	" "
**2.13**	Information from social media	" "

**2.14**	My own experience during medical studies	“ ”
	During your medical studies, did you hear any comments about general practice? Evaluate the comments you've heard from:	1. Negative<br>2. Very positive
**2.16**	Hospital specialists	“ ”
**2.17**	General practitioners	“ ”
**2.18**	Teachers	“ ”
**2.19**	Physician assistants	“ ”
**2.20**	Fellow students	“ ”
